# Diet Quality Compared to the Nutritional Knowledge of Polish, German, and Slovakian University Students—Preliminary Research

**DOI:** 10.3390/ijerph17239062

**Published:** 2020-12-04

**Authors:** Edyta Suliga, Elżbieta Cieśla, Sven Michel, Helena Kaducakova, Titus Martin, Grzegorz Śliwiński, Alexander Braun, Marcela Izova, Maria Lehotska, Dorota Kozieł, Stanisław Głuszek

**Affiliations:** 1 Institute of Health Sciences, Medical College, Jan Kochanowski University, 25-369 Kielce, Poland; edyta.suliga@ujk.edu.pl (E.S.); dorota.koziel@ujk.edu.pl (D.K.); 2 Institute of Ergonomics and Social Sciences, Faculty of Social Work, Health and Music, Brandenburg University of Technology Cottbus-Senftenberg, 03048 Cottbus, Germany; sven.michel@b-tu.de (S.M.); titus.martin@b-tu.de (T.M.); alexander.braun@b-tu.de (A.B.); 3 Department of Nursing, Faculty of Health, Catholic University in Ružomberok, 03401 Ružomberok, Slovakia; helena.kaducakova@ku.sk (H.K.); marcela.izova@ku.sk (M.I.); maria.lehotska@ku.sk (M.L.); 4 Institute of Biomedical Engineering, Technical University in Dresden, 01069 Dresden, Germany; grzegorz.sliwinski@tu-dresden.de; 5 Institute of Medical Sciences, Medical College, Jan Kochanowski University, 25-369 Kielce, Poland; sgluszek@wp.pl

**Keywords:** eating habits, young adults, nutritional knowledge

## Abstract

The eating habits of students differ significantly from those recommended by health practitioners. The aim of this study was to find differences related to diet quality and knowledge on nutrition among Polish, German, and Slovakian students as well as to examine which factors differentiate the diet quality of students from these three countries. The study was conducted on a group of 394 university students from Poland, Germany, and Slovakia. The assessment of diet quality and knowledge on food and nutrition was done with the use of the Dietary Habits and Nutrition Beliefs Questionnaire. The diet of German students was characterized by a significantly higher consumption of legume-based foods, vegetables, and fruit compared to Polish students and Slovakian participants (*p* < 0.001). The diet of the Poles was characterized by a high consumption of cured meat, smoked sausages, hot dogs, white bread and bakery products, butter, fried foods, and energy drinks. The most important factors significantly associated with diet quality involved the country, place of residence, Body Mass Index (BMI), physical activity, and time spent watching TV or using a computer. Polish students were characterized by the highest level of knowledge on food and nutrition (*p* < 0.001). However, it was not reflected in their diet. The authorities of universities should aim to provide students with access to canteens on campuses which would offer the possibility of consumption of both affordable and healthy meals.

## 1. Introduction

Eating habits involve a person’s behavior related to the choice of food and its consumption. This behavior is an effect of the interaction between genetic, demographic, socioeconomic, and cultural factors [1,2,3,4]. Therefore, food intake in various countries can differ due to social and economic factors, cultural traditions, and different food access [1,5]. On the other hand, the so-called “westernization” of a diet has been observed all around the world for many years [6,7,8]. It involves moving away from eating habits traditional for a given region and moving towards an increased consumption of sugar and sweets, sweetened beverages, fast food, red meat, and processed food. The “westernization” of a diet is particularly often observed during the transition from adolescence to adulthood [9,10,11,12] and is connected with a high risk of the occurrence of chronic diseases, e.g., obesity, metabolic syndrome, nonalcoholic fatty liver disease, type 2 diabetes, cardiovascular diseases, and several tumors [13]. However, the health consequences of poor diet quality do not appear immediately but after many years [14], which could explain why young people do not always care about following the principles of a healthy diet.

For students, commencing studies at university usually means the beginning of independence and the shaping of a students’ own lifestyle. This is the time when previously acquired patterns, related to meeting dietary needs, are fixed or new patterns of behavior are formed [15]. A change of place of residence (leaving the family home), an unstable economic situation, an increased educational workload, lack of time, and inadequate knowledge on the principles of a healthy diet often lead to students’ eating habits being significantly different from a good balanced diet [16,17,18,19,20,21,22].

So far, it has not been satisfactorily explained to what extent students apply nutritional knowledge in their daily nutritional practices. Several studies stated that a higher level of knowledge was associated with more healthy food choices [23,24]. However, some researches have not noted such associations [25]. Rivera Medina et al. found that dietary habits were associated with nutritional knowledge but not with knowledge on cooking methods and techniques [26].

The aim of the study was to examine differences related to diet quality and the level of knowledge among Polish, German, and Slovakian students as well as to find out which factors differentiate the diet quality of students from these three countries. Poland, Slovakia, and the former GDR (German Democratic Republic) are neighboring countries where a similar systemic, economic, and social transformation took place, which began in the 1980s. Currently, however, these countries differ in terms of the level of economic development as well as social development, measured by the value of the Human Development Index (HDI). This fact may affect the diet quality of the inhabitants of these countries.

## 2. Material and Methods

The study was conducted in the period from November 2019 to March 2020 on a group of 394 university students from Poland, Germany, and Slovakia. They were students from the Medical College of Jan Kochanowski University in Kielce in Poland (*n* = 135, 34.26%); the Faculty of Social Work, Health, and Music, The Brandenburg University of Technology Cottbus-Senftenberg in Germany (*n* = 107, 27.16%); and the Faculty of Public Health of The Catholic University in Ružomberk in Slovakia (*n* = 152, 38.58%). Of all the participants, 70.81% were females. Students were recruited via information about the study given at classes and lectures. They were informed about the aim of the study and about the fact that their participation was voluntary and that withdrawal was possible at every stage. The response rates were 92%, 95%, and 96% for Polish, German, and Slovak students, respectively. The study was approved by The Bioethics Committee of the Faculty of Medicine and Health Sciences of Jan Kochanowski University in Kielce (No 10/2015 dated 8 April 2015).

The evaluation of eating habits and knowledge on food and nutrition was performed with the use of the Dietary Habits and Nutrition Beliefs Questionnaire devised in Poland for people aged 15–65 years old (KomPAN) [27]. Questionnaire validation conducted on the Polish population revealed that its repeatability was from moderate to very good [28]. The cross-classification (test–retest) agreement of classification into the same category obtained for the self-administered questionnaire ranged from 63.8% to 84.7%. Kappa statistics in the group of healthy individuals ranged from 0.55 to 0.78. The questionnaire was translated from Polish into German and Slovakian by two independent translators. Next, both language versions were retranslated into Polish and the most linguistically suitable version of the questionnaire was established.

The first part of the questionnaire includes questions characterizing eating habits. The second part, which consists of 32 questions, evaluating the frequency of food consumption in six consumption categories (“never”, 1–3 times a month, once a week, a few times a week, once a day, “a few times in a day”). In this study, only 24 questions were used to calculate dietary indices.

For a comprehensive evaluation of diet, two indicators were calculated, one of which was related to food having potential health benefits, the so-called “Pro-Healthy Diet Index”, and the second which was related to food bad for health, the “Non-Healthy Diet Index”. The healthy index was calculated by summing up the frequency of consumption (times/day) of the following 10 food groups: vegetables, fruit, legume-based foods, fish, white meat, milk, fermented milk drinks, fresh cheese curd products, wholemeal (brown) bread/bread rolls, buckwheat, oats, wholegrain pasta, or other coarse-ground groats. The index of unhealthy diet was calculated by summing up the frequency of consumption (times/day) of the following 14 food groups: white bread and bakery products, white rice, white pasta, fine-ground groats, fast foods, fried foods, butter, lard, cheese (including processed cheese and blue cheese), red meat, cured meat, smoked sausages, hot dogs, tinned (jar) meats, sweets, sweetened carbonated or still drinks, energy drinks, and alcoholic beverages. The indices calculated as a sum of times/day were interpreted as follows: low (0–6.66), moderate (6.67–13.33), and high (13.34–20.00) Pro-Healthy Diet Index and low (0–9.33), moderate (9.34–18.66), and high (18.67–28.00) Non-Healthy Diet Index.

The third part involves statements on food and nutrition of a diversified level of difficulty, which allowed us to differentiate between students of inadequate, satisfactory, and good nutritional knowledge. The last part of the questionnaire includes questions related to the respondents’ lifestyle (smoking, physical activity, and time spent watching TV or using a computer) and their sociodemographic characteristics. Declared data on height and body mass were used for BMI calculation (in 386 among 394 participants—due to incomplete data).

The level of knowledge on food and nutrition was established on the basis of answers given to 25 statements included in the questionnaire [27]. One point was given for a correct answer to each of the 25 statements. The score was interpreted in the range 0–8 points as inadequate, 9–16 points as satisfactory, and 17–25 points as a good level of nutrition knowledge.

### Statistical Analysis

The statistical package STATISTICA 13.3 Software (Tibco Software Inc., Palo Alto, CA, USA) was used for analysis of the collected material. For quantitative variables (BMI, level of knowledge on food and nutrition in points, and values of indices of healthy and unhealthy diet), the arithmetic means and standard deviations were calculated. Due to a lack of normality of distributions, for comparisons of quantitative variables in three countries, the nonparametric Kruskal–Wallis test was applied, and in the case of significant correlations, the post hoc Dunn test was used. For categorical variables (sex, place of residence, financial situation, BMI category, health, diet, smoking, number of hours spent watching TV or using a computer, and physical activity), number and percentages were calculated. The distributions of these variables in the three countries were compared with the use of the nonparametric Chi-Square Test. In order to determine the strength of influence of independent variables (sex, place of residence, financial situation, BMI, level of knowledge on food and nutrition, number of hours spent watching TV or using a computer, and physical activity) on dependent variables (i.e., values of healthy and unhealthy diet in the student group altogether), Generalized Linear and Non Linear Regression (GLZ) analyses were performed using the identity method. Departure from linearity was assessed using the Wald test for nonlinearity. Sex was excluded from the analysis due to its insignificant correlation with healthy (r = 0.03; *p* = 0.529) and unhealthy diet (r = 0.09; *p* = 0.054). The first stage involved creating univariate models for each diet index. Subsequently, stepwise regression, backward elimination regression, was applied in order to build multivariate models aimed at finding the most important predictors for each diet index separately. In both cases, β coefficients were calculated with the use of the identity binding function. Reference points were adopted as follows: place of residence—big cities (>100,000 inhabitants), financial situation—above average, high physical activity, and the shortest time spent in front of TV/computer (less than 2 h). In the case of the “country” variable, Germany was adopted as a reference due to the highest Human Development Index. In 2018, it equaled 0.939 in Germany, whereas in Poland and Slovakia, they were 0.872 and 0.857, respectively [29]. Statistical significance was adopted at the level of *p* ≤ 0.05.

## 3. Results

The average age of the students was 21.52 ± 3.22 years (Table 1). Students from Poland and Slovakia were mostly inhabitants of rural areas, whereas German students mostly came from small towns (<20,000 inhabitants). Most of the participants described their financial situation as average (77.26%). The largest group of participants declaring their situation below average was from Germany (32.69%). In over 20% of subjects, overweight was diagnosed (BMI ≥ 25 kg/m^2^), including in 3.87%, obesity (BMI ≥ 30 kg/m^2^). Among the participants from the 3 countries, there were no significant differences in the prevalence of body mass deficiency or excess, smoking, health condition, and following a diet at present. German students spent the longest time in number of hours spent watching TV or using a computer. However, they also declared a high level of physical activity (43.93%). The participants from Poland declared the lowest level of physical activity (33.33%).

Among the subject students, only a few followed a very healthy or very unhealthy diet, and among the participants from the three countries, there were no significant differences between the healthy (Table 2) and unhealthy diet (Table 3) indices listed in the questionnaire. However, the sums of frequencies of consumption of food with a potentially beneficial health effect (times/day) differ among students from the 3 countries and were significantly lower among the participants from Poland compared to 2 other countries (*p* < 0.001). Moreover, a detailed analysis of frequency of the consumption of separate food groups included in the construction of the index of healthy diet revealed that German students consumed legumes, vegetables, and fruit more often than Poles and Slovaks, whereas they consumed less frequently white meat. Furthermore, German students more often than Slovaks consumed whole grain products. Total consumption of food of a potentially negative effect on health (according to the “Non-Healthy Diet Index”) also differed significantly (*p* < 0.001), and the scores were significantly higher in the group of Polish students compared to German or Slovakian ones. The analysis of frequencies of consumption of particular food groups of a potentially negative effect on health showed that Polish students consumed white bread and butter and drank energy drinks significantly more often. Slovakian students consumed white pasta and white rice and drank alcohol less frequently than German students and consumed fried food, cheeses, and cured meat least frequently among the 3 countries, whereas the most often consumed was lard. They also consumed red meat more often than Poles and tinned food more often than Germans. There were no significant differences between the 3 countries in the consumption of fast food, sweets, and sweetened drinks.

Statistically significant differences were related to the level of knowledge on nutrition, evaluated on the basis of the answers given to the 25 statements included in the questionnaire concerning food and nutrition (*p* < 0.001) (Table 4). Students from Poland obtained the most points (12.91 ± 2.70), whereas students from Slovakia got the smallest number of points (10.24 ± 3.23).

Univariate generalized linear regression (GLZ) showed that the following factors had an effect on the Pro-Healthy Diet Index: country, place of residence, and physical activity (Table 5). Students from Poland were characterized by a significantly lower intensity of healthy diet index compared to students from Germany (β = −1.50; *p* = 0.035). Students from rural environments less often followed a healthy diet (average by 1.48 point) compared to students from big cities (over 100 thousand inhabitants). Participants with low physical activity scored 3.42 points regarding following a healthy diet, and the score was lower compared to participants with high physical activity. Multivariate regression analysis confirmed a significant and negative effect of low physical activity on the value of the Pro-Healthy Diet Index. Students with low physical activity showed a lower intensity of a healthy diet by 3.17 points compared to students with high physical activity (β = −3.17; *p* = 0.001). A significant correlation was found between BMI and Pro-Healthy Diet Index. Along with the increase of BMI by 1 kg/m^2^, the value of the indicator increased by 0.44 points (*p* = 0.006).

Univariate generalized linear regression (GLZ) revealed a significant influence of the students’ country, place of residence, subjective opinion on financial situation, and physical activity on the value of the Non-Healthy Diet Index (Table 6). Compared to students from Germany, students from Poland had a significantly higher unhealthy diet index (β = 2.68; *p* < 0.001), whereas Slovakian students a significantly lower one (β = −1.21; *p* = 0.023). The following factors increased the score on the Non-Healthy Diet Index: being a resident of a rural area, having an average financial situation with regard to the student’s family, and having low physical activity. In contrast, high economic status and high physical activity significantly decreased the scores of the Non-Healthy Diet Index. Along with the increase of the number of points by 1 obtained for the knowledge on food and nutrition, there was an increase of unhealthy diet index by 0.44 points. However, multivariate linear regression analysis did not confirm this correlation. The most important predictors for the Non-Healthy Diet Index included country, place of residence, time spent watching TV or using a computer, and physical activity. A much higher score on the index was found among Polish students compared to German participants (β = 2.29; *p* < 0.001), countryside inhabitants compared to residents of big cities (β = 2.76; *p* = 0.001), and those who spent 6–8 h watching TV or using a computer, compared to those spending less than 2 h watching TV or using a computer (β = 2.94; *p* = 0.028). A similar value of the coefficient (β = 2.97) was found in participants spending >10 h watching TV or using a computer. However, it did not exceed the level of statistical significance (*p* = 0.411). Average physical activity turned out to be a predictor significantly decreasing the scores on the Non-Healthy Diet Index compared to high physical activity (β = −1.27; *p* = 0.019).

## 4. Discussion

The consumption of food of a potentially negative effect on health differed significantly between countries, and the scores were significantly higher in the group of Polish students compared to German or Slovakian ones. There were no differences in the consumption of products such as fish, milk and fermented milk drinks, fresh cheese curd products, sweets, sweetened beverages, and fast food. It indicates similarities in the diets of students from these 3 countries. Similar tendencies related to the diet quality of adolescents and university students have also been reported in other studies in recent years [11,30]. It should also be noted that the subject students were from neighboring countries; thus, probably also because of this fact, cultural differences concerning eating habits were not very large. Despite several similarities, the diets of students in the 3 countries were different as far as the consumption of some products is concerned. The diet of German students was of the healthiest nature. It was characterized by a significantly higher consumption of legume-based foods, vegetables, and fruit compared to Polish students and Slovakian participants. The unhealthiest was the diet of the Poles. It was characterized by a high consumption of cured meat, smoked sausages, hot dogs, white bread and bakery products, butter, fried foods, and energy drinks.

The highest level of knowledge on food and nutrition among the subject students was characteristic for the participants from Poland. However, it was not connected with their healthier diet. In the study involving Spanish students, it was also found that, even if students presented a better knowledge on nutrition, no positive changes were found in their eating habits or health-seeking behavior [31]. Better knowledge among students does not always entail a healthier diet. The cause of the abovementioned situation could be, in our opinion, the lack of canteens on the campuses of the Polish and Slovakian universities, which could offer easily accessible meals to students, as in the case of the German university. The results of various studies indicate that factors facilitating a healthy diet can be better knowledge and education on food, meal planning, engagement in preparation of meals, and physical activity [15,20].

Factors significantly correlated with the Pro-Healthy Diet Index in this study involved only physical activity and BMI. Students with a higher BMI were characterized by higher scores on the Pro-Healthy Diet Index. The results of the study confirm that a higher BMI is usually an effect of higher calorie consumption and/or lower physical activity [32,33]. The application of the Dietary Habits and Nutrition Beliefs Questionnaire does not allow us to calculate a diet’s energy value. Bearing in mind that the conducted study was cross-sectional, we cannot determine precisely a cause-and-effect relationship between food consumption and BMI. Therefore, it is possible that students with a higher BMI tried to modify their diet by consuming more products of a potential healthy nature. The effect of these changes can be a positive correlation between BMI and the Pro-Healthy Diet Index found in the study. Moreover, BMI is not a very precise measure of adipose tissue content, and high values of the indicator can result from big muscle mass [34,35].

Students with low physical activity had lower scores on the healthy diet index. It is in compliance with the results obtained by other authors showing that pro-health behaviors tend to accumulate [36,37]. Studies conducted in Spain [38,39] and in Cyprus [40] confirmed that more physically active students showed greater adherence to the Mediterranean diet compared to those less active.

The most important factors significantly related to the Non-Healthy Diet Index were country, place of residence, physical activity, and time spent watching TV or using a computer. Significantly higher scores on the unhealthy diet index was found among Polish students compared to German students, inhabitants of rural areas compared to residents of big cities, and those who spent 6–8 h watching TV or using a computer compared to students spending less than 2 h watching TV or using a computer. However, participants showing average physical activity had lower scores on the Non-Healthy Diet Index compared to those with high activity (β = −1.31). The reason for a significantly higher scores on the unhealthy diet index found among Polish students compared to German participants could be, as mentioned earlier, the lack of a canteen at the campus offering easily accessible meals to students.

Higher scores on the unhealthy diet index were found among students from rural areas compared to those from big cities. This relationship is in compliance with the results presented in other publications, in which it was confirmed that, in many European countries, the diet of countryside residents differed from the principles of healthy eating habits compared to residents of cities [41,42,43,44,45]. The most frequent nutrition mistakes among people from rural areas included the preference of fast food; frequent consumption of sweets and a small number of meals in a day [41]; lower consumption of fish, milk and dairy products, wholemeal cereal products, vegetables, and fruit [43]; and excessive consumption of sugar [44,45] and salt [45]. Studies of adolescents aged 10 to 12 from Portugal showed that, in an urban environment, meat, fish, and fruit were consumed more often than in rural areas. Moreover, in a rural environment, fruit availability was more season-dependent and thus less diversified [46]. However, in some studies, an opposite association was found, e.g., Scottish 15-year-olds from rural areas have a healthier diet than adolescents from urban areas [47]. In Slovakia, more healthy behaviors were observed related to the prevalence of substance use among rural girls compared to urban girls [48]. The study conducted in Poland showed that adolescents from rural areas compared to those from an urban environment were characterized by higher physical activity and a smaller effect of stress on excessive food consumption [41]. Nutrition mistakes among people from rural areas usually are caused by an unfavorable economic and social situation in these areas, including a lower level of education, fewer possibilities to get a well-paid job, lower availability, and higher prices of healthy food [49,50,51,52].

Students with the highest physical activity had, as previously described, higher scores on the healthy diet index. However, it was also found that students with average physical activity were characterized by lower scores on the Non-Healthy Diet Index compared to those with high activity. This fact can indicate a greater diversity of products (both healthy and unhealthy) consumed by individuals with high physical activity. In studies conducted among children and adolescents, it was found that those who were more physically active—in order to meet increased energy expenditure—consumed more diversified food, and they were not always healthy products, as it may have been expected [53,54]. Therefore, it is possible that a similar phenomenon is present among the subject students. A positive correlation between a longer time spent in front of TV and computer and higher scores on the Non-Healthy Diet Index is compliant with the previously described tendency of the coexistence of various behaviors negative for health [36,37].

### Strengths and Limitations

A basic limitation is the fact that the selection of participants was not random. They volunteered to participate in the study; thus, we cannot rule out the possibility of participation bias. Participants in each country were from one academic institution each, which may limit the generalizability of our results. Over 70% of participants were females, which results from specific gender distributions in the departments where the study was conducted. However, the percentage of males was similar in each of 3 compared countries. Another limitation was the impossibility to calculate the energy value of diets of the subject students. Diet quality of participants could have been affected by other, unrecognized factors. A strength of the study is the application of the same standardized questionnaire of a precisely defined and repeatable procedure of analysis and interpretation of scores in each country. Study participants in all 3 countries studied at departments related to health knowledge (physiotherapy, public health, and nursing).

## 5. Conclusions

Among students from these three countries, only 2% had a very unhealthy or moderately unhealthy diet. None of the students surveyed could be classified as applying a very healthy diet, and 12.4% followed a moderately healthy diet.

The diet quality of students in the 3 countries were varied in a lot of ways. German students had the healthiest diet. It was characterized by the highest consumption of legume-based foods, vegetables, and fruit. The diet of Polish students was not compliant with the principles of healthy nutrition to the largest extent. It involved a high consumption of cured meat, smoked sausages, hot dogs, white bread and bakery products, butter, fried foods, and energy drinks.

The most important factors significantly associated with diet quality involved the country, place of residence, BMI, physical activity, and time spent watching TV or using a computer. The highest scores on the Pro-Healthy Diet Index were noted among students who were the most physically active and had a higher BMI. The highest scores on the Unhealthy Diet Index were found among Polish students compared to German participants, inhabitants of rural areas compared to residents of big cities, and those who spend more than 6–8 h watching TV or using a computer compared to those whose physical activity.

Polish students were characterized by the highest level of knowledge on food and nutrition. However, it was not reflected in their diet. The authorities of universities should aim to provide students with access to canteens on campuses, which would offer the possibility of consumption of both affordable and healthy meals.

## Figures and Tables

**Table 1:** Sociodemographic and health characteristics of subject students.

Variables	Categories	Total	Poland	Germany	Slovakia	*p* ^1^
Gender *n* (%)	Women	279 (70.81)	96 (71.11)	74 (69.16)	109 (71.71)	0.902
	Men	115 (29.19)	39 (28.89)	33 (30.84)	43 (28.29)	
Place of residence *n* (%)	Rural areas	170 (43.93)	61 (45.52)	26 (24.30)	83 (56.85)	**<0.001**
	Town <20,000 inhabitants	72 (18.60)	11 (8.21)	33 (30.84)	28(19.18)	
	Town: 20,000–100,000 inhabitants	75 (19.38)	20 (14.93)	28 (26.17)	27 (18.49)	
	City >100,000 inhabitants	70 (18.09)	42 (31.34)	20 (18.69)	8 (5.48)	
Financial situation *n* (%)	Below average	61 (15.76)	9 (6.67)	34(32.69)	18 (12.16)	**<0.001**
	Average	299 (77.26)	106 (78.52)	66 (63.46)	127 (85.81)	
	Above average	27 (6.98)	20 (14.81)	4 (3.85)	3 (2.03)	
BMI (kg/m^2^)	X ± SD	22.46 ± 3.86	22.00 ± 3.73	22.41 ± 3.80	22.91 ± 3.99	0.333
BMI (kg/m^2^), *n* (%) *n* = 388	<18.5	29 (7.47)	6 (5.94)	10 (8.55)	10 (6.58)	0.357
	18.5–24.9	278(71.65)	74(73.27)	80(68.38)	106 (69.74)	
	25.0–29.9	66 (17.01)	18 (17.82)	17 (14.53)	26 (17.11)	
	≥30.0	15 (3.87)	3 (2.97)	10 (8.55)	10 (6.58)	
Health status in comparison to other people, *n* (%)	Worse than others	43 (11.00)	16 (11.94)	10 (9.35)	17 (11.33)	0.764
	The same as others	275 (70.33)	89 (66.42)	78 (72.90)	108 (72.00)	
	Better than others	73 (18.67)	29 (21.64)	19 (17.76)	25 (16.67)	
Are you currently following a diet? *n* (%)	No	348 (88.32)	94 (87.85)	104 (88.89)	139 (91.45)	0.141
	Yes, as advised by my doctor for medical reasons	8 (2.03)	4 (2.96)	0 (0.00)	4 (2.63)	
	Yes, it was my personal decision	38 (9.64)	16 (11.85)	13 (12.15)	9 (5.92)	
Smoking *n* (%)	No	301 (76.59)	102 (76.12)	87 (81.31)	112 (73.68)	0.357
	Yes	92 (23.41)	32 (23.88)	20 (18.69)	40 (26.32)	
Hours/day spend watching TV or using a computer *n* (%)	Less than 2 h	113 (28.83)	49 (36.84)	12 (11.21)	52 (34.21)	**<0.001**
	from 2 to almost 4 h	142 (36.22)	46 (34.59)	38 (35.51)	58 (38.16)	
	from 4 to almost 6 h	82 (20.92)	27 (20.30)	34 (31.78)	21 (13.82)	
	from 6 to almost 8 h	44 (11.22)	7 (5.26)	19 (17.76)	18 (13.82)	
	from 8 to almost 10 h	8 (2.04)	3 (2.26)	3 (2.80)	2 (1.32)	
	More than 10 h	3 (0.77)	1 (0.75)	1 (0.93)	1 (0.66)	
Physical activity during time off *n* (%)	Low	80 (20.41)	45 (33.33)	11 (10.28)	24 (16.00)	**<0.001**
	Moderate	207 (52.81)	54 (40.00)	49 (45.79)	104 (69.33)	
	High	105 (26.79)	36 (26.67)	47 (43.93)	22 (14.67)	

^1^ Chi square test; bold indicates statistically significant results.

**Table 2:** Pro-Healthy Diet Index and frequency of consumption of food groups potentially beneficial for health (times/day).

“Pro-Healthy Diet Index” and Its Components	Total X ± SD *n* = 394	Poland X ± SD *n* = 135	Germany X ± SD *n* = 107	Slovakia X ± SD *n* = 152	*p*
Low, *n* (%)	345 (87.56)	123 (91.11)	93 (84.87)	129 (84.87)	0.270 ^a^
Medium, *n* (%)	49 (12.44)	12 (8.89)	23 (15.13)	12 (8.89)	
High, *n* (%)	0 (0.00)	0 (0.00)	0 (0.00)	0 (0.00)	
Sum of frequency of food consumption (times/day) ^1^	4.20 ± 2.02	3.92 ± 1.89	4.54 ± 1.96	4.20 ± 2.14	**0.029 ^b^**
Wholemeal (brown) bread/bread rolls ^2^	0.37 ± 0.40	0.40 ± 0.44	0.43 ± 0.41	0.31 ± 0.36	**0.015 ^b^**
Buckwheat, oats, wholegrain pasta, coarse-ground groats	0.32 ± 0.45	0.26 ± 0.33	0.32 ± 0.40	0.36 ± 0.57	0.357 ^b^
Milk	0.53 ± 0.53	0.52 ± 0.53	0.51 ± 0.53	0.56 ± 0.53	0.496 ^b^
Fermented milk drinks	0.38 ± 0.39	0.36 ± 0.42	0.33 ± 0.35	0.43 ± 0.40	0.054 ^b^
Fresh cheese curd products	0.21 ± 0.30	0.22 ± 0.27	0.22 ± 0.35	0.19 ± 0.28	0.201 ^b^
White meat ^3^	0.40 ± 0.37	0.46 ± 0.36	0.26 ± 0.26	0.45 ± 0.42	**<0.001 ^b^**
Fish	0.15 ± 0.23	0.13 ± 0.20	0.17 ± 0.26	0.15 ± 0.24	0.602 ^b^
legume-based foods ^4^	0.15 ± 0.18	0.11 ± 0.15	0.23 ± 0.24	0.12 ± 0.13	**<0.001 ^b^**
Fruit ^5^	0.88 ± 0.61	0.72 ± 0.52	1.06 ± 0.63	0.88 ± 0.64	**<0.001 ^b^**
Vegetables ^6^	0.82 ± 0.61	0.74 ± 0.57	1.01 ± 0.63	0.75 ± 0.61	**<0.001 ^b^**

^a^ Chi square test; ^b^ Kruskal–Wallis test; bold indicates statistically significant results. Post hoc Dunn tests scores: ^1^ Poland–Slovakia: *p* < 0.001; Poland–Germany: *p* < 0.001; Germany–Slovakia: *p* = 0.726; ^2^ Poland–Slovakia: *p* = 0.212; Poland–Germany: *p* = 0.958; Germany–Slovakia: *p* = 0.020; ^3^ Poland–Slovakia: *p* = 1.000; Poland–Germany: *p* < 0.001; Germany–Slovakia: *p* < 0.001; ^4^ Poland–Slovakia: *p* = 0.062; Poland–Germany: *p* < 0.001; Germany–Slovakia: *p* = 0.002; ^5^ Poland–Slovakia: *p* = 0.137; Poland–Germany: *p* = 0.033; Germany–Slovakia: *p* = 0.033; ^6^ Poland–Slovakia: *p* = 1.000; Poland–Germany: *p* = 0.001; Germany–Slovakia: *p* < 0.001.

**Table 3:** Non-Healthy Diet Index and frequency of consumption of food groups of a potentially adverse effect on health (times/day).

“Non-Healthy Diet Index” and Its Components	Total X ± SD *n* = 394	Poland X ± SD *n* = 135	Germany X ± SD *n* = 107	Slovakia X ± SD *n* = 152	*p*
Low, *n* (%)	386 (97.97)	130 (96.30)	105 (98.13)	151 (99.34)	0.388 ^a^
Medium, *n* (%)	7 (1.78)	4 (2.96)	2 (1.87)	1 (2.96)	
High, *n* (%)	1 (0.25)	1 (0.74)	0 (0.00)	0 (0.00)	
Sum of frequency of food consumption (times/day) ^1^	4.12 ± 2.22	4.86 ± 2.57	3.70 ± 1.90	3.77 ± 1.92	**<0.001 ^b^**
White bread and bakery products ^2^	0.63 ± 0.59	0.84 ± 0.66	0.46 ± 0.48	0.56 ± 0.54	**<0.001 ^b^**
White rice, white pasta, fine-ground groats ^3^	0.40 ± 0.37	0.40 ± 0.37	0.43 ± 0.27	0.37 ± 0.43	**0.004 ^b^**
Fast foods	0.13 ± 0.18	0.16 ± 0.23	0.11 ± 0.11	0.12 ± 0.16	0.110 ^b^
Fried foods ^4^	0.30 ± 0.29	0.38 ± 0.31	0.30 ± 0.23	0.23 ± 0.30	**<0.001 ^b^**
Butter ^5^	0.49 ± 0.58	0.75 ± 0.71	0.23 ± 0.34	0.43 ± 0.48	**<0.001 ^b^**
Lard ^6^	0.08 ± 0.23	0.05 ± 0.26	0.03 ± 0.12	0.14 ± 0.26	**<0.001 ^b^**
Cheese ^7^	0.37 ± 0.35	0.43 ± 0.41	0.48 ± 0.32	0.23 ± 0.27	**<0.001 ^b^**
Cured meat, smoked sausages, hot dogs ^8^	0.39 ± 0.41	0.57 ± 0.45	0.40 ± 0.44	0.22 ± 0.25	**<0.001 ^b^**
Red meat ^9^	0.20 ± 0.23	0.16 ± 0.20	0.21 ± 0.24	0.23 ± 0.24	**0.014 ^b^**
Tinned (jar) meats ^10^	0.05 ± 0.14	0.04 ± 0.14	0.02 ± 0.07	0.07 ± 0.18	**0.003 ^b^**
Sweets	0.62 ± 0.57	0.55 ± 0.55	0.61 ± 0.58	0.68 ± 0.59	0.094 ^b^
Sweetened carbonated or still drinks	0.24 ± 0.43	0.25 ± 0.46	0.17 ± 0.31	0.29 ± 0.46	0.122 ^b^
Energy drinks ^11^	0.09 ± 0.25	0.14 ± 0.29	0.07 ± 0.29	0.07 ± 0.17	**0.002 ^b^**
Alcoholic beverages ^12^	0.14 ± 0.23	0.13 ± 0.21	0.17 ± 0.18	0.13 ± 0.26	**0.003 ^b^**

^a^ Chi square test; ^b^ Kruskal–Wallis test; bold indicates statistically significant results. Post hoc Dunn tests scores: ^1^ Poland–Slovakia: *p* < 0.001; Poland–Germany: *p* < 0.001; Germany–Slovakia: *p* = 1.000; ^2^ Poland–Slovakia: *p* < 0.001; Poland–Germany: *p* < 0.001; Germany–Slovakia: *p* = 0.342; ^3^ Poland–Slovakia: *p* = 0.236; Poland–Germany: *p* = 0.475; Germany–Slovakia: *p* = 0.006; ^4^ Poland–Slovakia: *p* < 0.001; Poland–Germany: *p* = 0.430; Germany–Slovakia: *p* < 0.001; ^5^ Poland–Slovakia: *p* = 0.005; Poland–Germany: *p* < 0.001; Germany–Slovakia: *p* = 0.004; ^6^ Poland–Slovakia: *p* < 0.001; Poland–Germany: *p* = 1.000; Germany–Slovakia: *p* < 0.001; ^7^ Poland–Slovakia: *p* < 0.001; Poland–Germany: *p* = 0.244; Germany–Slovakia: *p* < 0.001; ^8^ Poland–Slovakia: *p* = 0.013; Poland–Germany: *p* < 0.001; Germany–Slovakia: *p* < 0.001; ^9^ Poland–Slovakia: *p* = 0.029; Poland–Germany: *p* = 0.090; Germany–Slovakia: *p* = 1.000; ^10^ Poland–Slovakia: *p* = 0.185; Poland–Germany: *p* = 1.000; Germany–Slovakia: *p* = 0.024; ^11^ Poland–Slovakia: *p* = 0.022; Poland–Germany: *p* = 0.023; Germany–Slovakia: *p* = 1.000; ^12^ Poland–Slovakia: *p* = 0.315; Poland–Germany: *p* = 0.368; Germany–Slovakia: *p* = 0.005.

**Table 4:** Nutrition knowledge level (answers to the statements concerning food and nutrition).

Nutrition Knowledge Level (points)	Total	Poland	Germany	Slovakia	*p* ^1^
	*n* (%)	*n* (%)	*n* (%)	*n* (%)	
Insufficient 0–8	74 (18.78)	7 (5.19)	24 (22.43)	43 (28.29)	**<0.001**
Sufficient 9–16	299 (75.89)	115 (85.19)	76 (71.03)	108 (71.05)	
Good 17–25	21 (5.33)	13 (9.63)	7 (6.54)	1 (0.66)	
X ± SD	11.37 ± 3.32	12.91 ± 2.70	11.01 ± 3.23	10.24 ± 3.23	**<0.001**

^1^ Chi square test; bold indicates statistically significant results.

**Table 5:** Univariate and multivariate generalized linear regression analysis for Pro-Healthy Diet Index.

Variables	Categories	Univariate GLZ Analysis for Pro-Healthy Diet Index				Multivariate GLZ for Pro-Healthy Diet Index			
		β	95% CI	SE	*p*	β	95% CI	SE	*p*
Country	Germany	ref.							
	Poland	**−1.50**	**−2.89–−0.10**	**0.71**	**0.035**				
	Slovakia	−0.01	−1.44–1.27	0.69	0.904				
Place of residence	City >100,000 inhabitants	ref.							
	Town: 20,000–100,000 inhabitants	0.55	−1.33–2.44	0.96	0.564				
	Town <20,000 inhabitants	1.03	−0.87–2.95	0.98	0.288				
	Rural areas	**−1.48**	**−2.96–−0.01**	**0.75**	**0.049**				
Financial situation	Above average	ref.				ref.			
	Average	0.50	−1.21–2.20	0.87	0.567	1.65	−0.32–3.63	1.01	0.101
	Below average	−0.49	−2.63–1.66	1.09	0.656	−2.43	−5.15–0.28	1.36	0.079
BMI	kg/m^2^	0.26	−0.03–0.55	0.15	0.084	**0.44**	**0.12–0.76**	**0.16**	**0.006**
Knowledge about nutrition	points	0.17	−0.13–0.47	1.18	0.278	0.14	−0.20–0.59	0.16	0.411
Hours/day spend watching TV or using a computer	less than 2 h	ref.							
	from 2 to almost 4 h	−1.38	−4.06–1.30	1.37	0.312				
	from 4 to almost 6 h	0.07	−2.06–2.98	1.49	0.960				
	from 6 to almost 8 h	2.68	−0.66–6.01	1.70	0.116				
	from 8 to almost 10 h	1.09	−5.00–7.17	3.11	0.727				
	more than 10 h	1.05	−4.17–2.06	1.59	0.507				
Physical activity during time off	High	ref.				ref.			
	Moderate	0.64	−0.65–1.94	0.94	0.331	−0.32	−1.84–1.21	0.93	0.683
	Low	**−3.42**	**−5.03–−1.80**	**0.83**	**<0.001**	**−3.17**	**−5.00–−1.33**	**0.93**	**0.001**

β—beta coefficient; SE—standard error; Numbers in bold indicates statistically significant results.

**Table 6:** Univariate and multivariate generalized linear regression analysis for the Non-Healthy Diet Index.

Variables	Categories	Univariate GLZ for Non-Healthy Diet Index				Multivariate GLZ for Non-Healthy Diet Index			
		β	95% CI	SE	*p*	β	95% CI	SE	*p*
Country	Germany	ref.				ref.			
	Poland	**2.68**	**1.61–3.75**	**0.55**	**<0.001**	**2.29**	**1.11–3.47**	**0.60**	**<0.001**
	Slovakia	**−1.21**	**−2.25–−0.16**	**0.53**	**0.023**	0.10	−1.02–1.23	0.58	0.856
Place of residence	City >100,000 inhabitants	ref.				ref.			
	Town: 20,000–100,000 inhabitants	−1.32	−2.82–−0.17	0.76	0.428	−0.66	−2.21–0.90	0.79	0.408
	Town <20,000 inhabitants	−0.60	−2.07–0.88	0.75	0.083	−0.12	−1.59–1.35	0.75	0.873
	Rural areas	**3.76**	**2.25–5.27**	**0.77**	**<0.001**	**2.76**	**1.17–4.35**	**0.81**	**0.001**
Financial situation	Above average	ref.							
	Average	**−1.42**	**−2.75–−0.09**	**0.68**	**0.037**				
	Below average	−0.67	−2.35–1.00	0.85	0.432				
BMI	kg/m^2^	−0.04	−0.27–0.19	0.12	0.741				
Knowledge about nutrition	points	**0.25**	**0.02–0.49**	**0.11**	**0.037**				
Hours/day spend watching TV or using a computer	Less than 2 h	ref.				ref.			
	from 2 to almost 4 h	−0.69	−2.79–1.42	1.08	0.524	−0.37	−2.38–1.64	1.03	0.718
	from 4 to almost 6 h	−0.21	−2.50–2.08	1.17	0.858	0.11	−2.10–2.31	1.13	0.924
	from 6 to almost 8 h	1.65	−0.98–4.28	1.34	0.219	**2.94**	**0.32–5.56**	**1.34**	**0.028**
	from 8 to almost 10 h	−3.83	−8.62–0.97	2.45	0.118	−3.60	−8.14–0.94	2.31	0.120
	more than 10 h	4.32	−3.15–11.79	3.81	0.257	2.97	−4.12–10.07	0.67	0.411
Physical activity during time off	High	ref.				ref.			
	Moderate	**−2.00**	**−3.03–−0.97**	**0.52**	**0.000**	**−1.27**	**−2.34–−0.21**	**0.57**	**0.019**
	Low	**1.91**	**0.63–3.20**	**0.65**	**0.003**	1.14	−0.18–2.48	0.68	0.091

β—beta coefficient; SE—standard error; Bold indicates statistically significant results.
